# CHEK2 1100delC is prevalent in Swedish early onset familial breast cancer

**DOI:** 10.1186/1471-2407-7-163

**Published:** 2007-08-17

**Authors:** Sara Margolin, Hans Eiberg, Annika Lindblom, Marie Luise Bisgaard

**Affiliations:** 1 Department of Oncology, Karolinska University Hospital at Södersjukhuset, Stockholm, Sweden; 2 Department of Medical Biochemistry and Genetics, Panum Institute 24.4, Copenhagen University, Copenhagen, Denmark; 3 Department of Molecular Medicine and Surgery, Karolinska Institute, Stockholm, Sweden; 4 Medical Genetics Clinic, Panum Institute 24.4, Copenhagen University, Copenhagen, Denmark

## Abstract

**Background:**

A truncating variant, 1100delC, in check point-kinase CHEK2, has been identified as a risk factor for familial and sporadic breast cancer. The prevalence in healthy non-breast cancer cases is low and varies between populations.

**Methods:**

We analyzed the prevalence of *CHEK2 *1100delC in 763 breast cancer patients with a defined family history and 760 controls from the Stockholm region. The breast cancer patients originated from; a population-based cohort (n = 452) and from a familial cancer clinic (n = 311), the detailed family history was known in both groups.

**Results:**

The variant was found in 2.9% of the familial cases from the population-based cohort and in 1.9% from the familial cancer clinic. In total 2.2% of the patients with a family history of breast cancer carried the variant compared to 0.7% of the controls (p = 0.03). There was no increased prevalence in sporadic patients (0.3%). The variant was most frequent in young familial patients (5.1% of cases ≤45 years, p = 0.003). The mean age at diagnosis of variant carriers was 12 years lower than in non-carriers (p = 0.001).

**Conclusion:**

In conclusion, *CHEK2 *1100delC exists in the Swedish population. The prevalence is increased in familial breast cancer and the variant seems to influence age at onset.

## Background

Apart from gender, family history is the most important risk factor for breast cancer. Mutations in the known high-risk genes *BRCA1, BRCA2, p53, ATM *and *PTEN *account for less than 25% of the familial risk for breast cancer while the remainder are still genetically unexplained despite large efforts in research [1]. A polygenic model with variations in several loci, each contributing a modest independent risk has been shown to best explain the residual non *BRCA1/2 *aggregation of breast cancer, and the effect of low penetrant genes may also at least partly explain sporadic breast cancer [2,3]. However, there are few conclusive results on variants in candidate low-penetrant genes even though a large number of case-control studies, most relatively small in sample size, have been performed [4]. Association studies on variants conferring modest risks require large sample sizes; the study size is however also influenced by the variant frequency. In general unselected breast cancer cases are used for association studies, but the study size can be reduced by selecting cases enriched for genetic susceptibility such as patients with family history or bilateral cases [5]. A rare truncating variant in *CHEK2 *(1100delC), a G2 checkpoint-kinase, that is involved in cell cycle control and DNA-repair, was identified as a risk factor for breast cancer in two independent studies using this approach [6-8]. The variant frequency was assessed in high-risk familial non *BRCA1/2 *cases and healthy controls, and both studies were highly significant despite the fact that the variant is rare (1% or less in normal population) and confers a relative risk of around 2 [7,8]. More than 600 unselected cases were tested in each study but no significant difference in allele frequency between unselected cases and controls was detected [7,8]. The variant was suggested to be a low-penetrance gene as described in the polygenic model but the possibility of a role as a modifier of as – yet unknown high-risk gene/s has also been proposed [7-9]. A subsequent collaboration study of more than 10 000 cases and controls has demonstrated a doubled risk also in unselected cases carrying the variant [10]. In the present study we analyse the importance of the variant among breast cancer patients with a known familial disposition compared to sporadic cases and controls. Furthermore, we evaluate the significance of the variant as a modifier gene versus a low-penetrance gene.

## Methods

### Material

#### Familial Risk Cohort

311 independent familial cases collected at the Department of Clinical Genetics at Karolinska Hospital, Stockholm were used for the study. These patients had either been referred due to a breast cancer diagnosis *and *a family history of breast cancer or had been collected as part of previous research on familial breast cancer [11]. All cases had proceeded through genetic counseling and almost all (>90%) had been screened negative for BRCA1/2 mutations (those who met the current criteria for screening and the majority of the others as part of previous research to define criteria for screening). BRCA1 and BRCA2 mutations are rare in the Stockholm region and in previous studies only 1% mutations in each gene were detected in familial breast cancer if not using age criteria [12,13].

For the Familial Risk cohort only age at diagnosis was available (missing in 16 patients).

#### Population-based cohort

Patients with a surgically treated primary invasive breast cancer admitted to the Department of Oncology at Huddinge Hospital and Söder Hospital (covering the population of southern Stockholm of 850 000 people) from October 1998 to May 2000 were asked participate in a study on genetic risk factors from breast cancer [14]. Family history, age at diagnosis, hormone receptor status and histology of the tumor were obtained from all cases and the median follow-up was 5 years. This cohort consists of 489 patients in total and 456 were used in this study due to logistic reasons. The samples had previously been screened for mutations in exon 11 of BRCA1 where more than 70% of the mutations, including four founder mutations, identified in the Stockholm region are found [14]. Four cases with known *BRCA1 *or *BRCA2 *mutations were excluded from the study.

Cases with at least one 1^st ^or 2^nd^-degree relatives with breast cancer in addition to the proband, regardless of age, were classified as familial breast cancer. In the Familial risk cohort 168 cases had one 1^st ^or 2^nd ^degree relative, while the remainder had more than one 1^st ^or 2^nd ^degree relative. In the population-based cohort 104 cases had one relative and 35 more than one relative with the disease.

The mean age at diagnosis in the Familial risk cohort was 54 years (24–92 years). In the Population-based cohort the mean age was 60 years (27–88 years) and there was no statistically significant difference between familial and sporadic cases (59 and 61 years respectively).

As controls we used DNA from 760 geographically matched blood-donors of mixed gender collected as a control material for association studies at Karolinska University Hospital, Stockholm, Sweden.

The Ethical Committee at the Karolinska Institute approved the study.

### Methods

Genotyping of the most common mutation in the *CHEK2 *gene (1100delC) was performed on controls and patients by PCR with a primer set that is specific for the mutation (M-CHEK2del100C-F: 5'-gca aag aca tga atc tgt aaa gtc-3' M-CHEK2del100C-R: 5'-aaa tct tgg agt gcc caa aat aat-3' and a primer set specific for the wild type allele: (W-CHEK21100delC-F: 5'-gca aag aca tga atc tgt aaa gtc-3' and 3' W-CHEK21100delC-R: 5'-aaa tct tgg agt gcc caa aat cag-3' resulting in 184 base pair products. DNA amplifications were carried out in a 20 μl volume containing 1 × Ampliqon III standard buffer (Ampliqon ApS, Copenhagen), 125 μM of each dNTP, 4 pmol of each primer, 0.5 units of Amplicon III Taq Polymerase (Ampliqon ApS, Copenhagen), and 25 – 50 ng of template DNA. The PCR conditions were: initial denaturation at 95°C for 5 min. hereafter 40 cycles at 95°C for 20 sec, 59°C for 20 sec and 72°C for 20 sec followed by 5 min. of extension at 72°C.

The two reactions were run separately each in multiplex with a control PCR (Control-F: 5'-gtc aaa gcc acc agt tac agt-3' and Control-R: 5'-ttc ccc acc act tta ctg ac-3') resulting in a product of 309 base pairs on chromosome 4. The products were separated on 2.1% SeaKem LE Agarose (Cambrex, Bio Science Rockland, Inc., Rockland, ME, USA) gels. In order to examine for homozygosity for the variant, PCR for wild type allele and control were performed in cases with the CHEK2 1100CdelC variant.

### Statistical analysis

Fisher's exact test was used to compare categorical data. Continuous data were compared using the two-sample t-test.

## Results

The prevalence of *CHEK2 *1100delC was 1.9% in the familial risk Cohort and 1.1% in the population-based cohort compared to 0.7% in the controls (p = 0.09 and 0.51 respectively). 2.2% of all the familial cases carried the variant (p = 0.03), corresponding to an odds ratio of 3.4 (95% CI 1.2–10.1). (Table 1). Only one of the sporadic cases carried the variant (0.3%). There was an increasing frequency of the variant with decreasing age at diagnosis in the familial patients (Table 2).

**Table 1:** Prevalence of CHEK2 1100delC in sporadic and familial breast cancer and controls

	**CHEK2 1100delC+/total tested**	**p-value^1^**
**Familial Risk Cohort**	**6/311 (1.9%)**	**0.09**
**Population-based Cohort**	**5/452 (1.1%)**	**0.51**
Sporadic breast cancer	1/313 (0.3%)	0.68
Familial Breast Cancer	4/139 (2.9%)	0.04
**All Familial patients**	**10/450 (2.2%)**	**0.03**
**Controls**	**5/760 (0.7%)**	

^1^P-values were calculated with Fisher's test for association

**Table 2:** Prevalence of CHEK2 1100delC in familial breast cancer according to age at diagnosis compared to controls (Familial Risk Cohort and Population-based cohort, age at diagnosis missing in 16 patients)

**Age at diagnosis, years**	**CHEK2 1100delC+/total tested**	**p-value^1^**
**<45**	5/98 (5.1%)	0.003
**46–55**	4/148 (2.7%)	0.04
**>55**	1/188 (0.5%)	1.0

^1^P-values were calculated with Fisher's test for association

The mean age at diagnosis of variant carriers was 12 (familial risk cohort) and 10 (population-based cohort) years lower in carriers than in non-carriers; 42 (24–55) vs. 54 years in familial risk cohort (p = 0.01) and 50 years (38–65) versus 60 years in the population-based cohort (p = 0.06). If the groups were combined the difference in mean age at onset was 12 years (46 vs. 58 years, p = 0.001).

**Figure 1:**
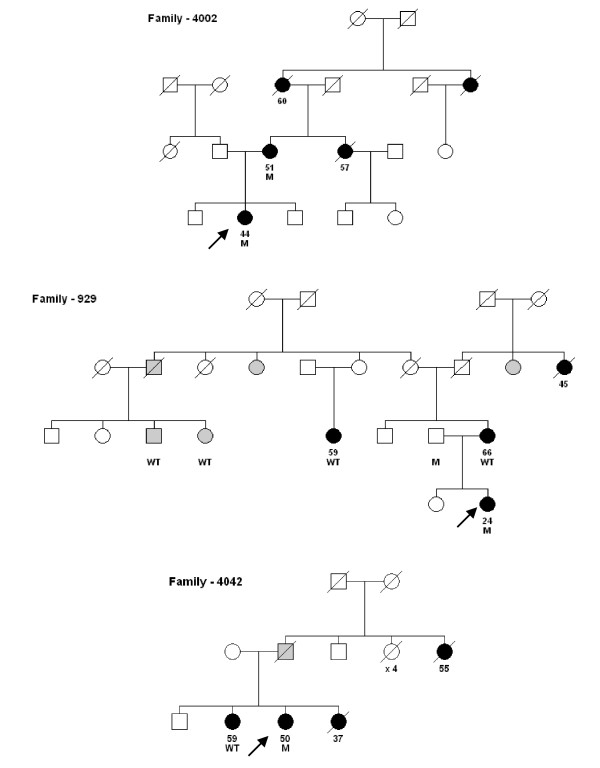
Pedigrees of families, arrow = index case. Black symbols are breast cancer cases; gray symbols are cases with any other cancer and the number below the symbols are age at diagnosis. WT = CHEK2 1100delC mutation, M = CHEK21100delC carrier.

In three families of carriers from the Familial Risk cohort, DNA was available for more family members. In two of these families, the mutation carrier status did not segregate with disease (Figure 1).

The five heterozygotes in the Population-based cohort all had unilateral ductal breast cancer without recurrence or secondary malignancies with 6 years follow-up.

Only one CHEK2 1100delC carrier did not have a family history of breast cancer (Table 3).

**Table 3:** Family history of the CHEK2 1100delC carriers (dr = degree relative/s)

**Family number and age at diagnosis**	**Breast Cancer**	**Other malignancies**
102, 65	Five 1^st ^dr including one male, age 40–67	One 1^st ^dr with cancer uteri
195, 54		One 1^st ^dr with brain tumour
510, 41	One 2^nd ^dr	
764, 52	One 1^st ^dr, age 28	
875, 38	One 1^st ^dr, age 36	One 1^st ^dr with non-Hodgkin lymphoma One 2^nd ^dr with colon cancer
929, 24	One 1^st ^dr, age 66 One 3^rd ^dr, age 45 One 4^th ^dr, age 59	One 3^rd ^dr with pancreatic cancer, Two 3^rd ^dr with cancer of the abdomen
1902, 55	One 2^nd ^dr, age 75	One 1^st ^dr with gyn. malignancy One 2^nd ^dr with cancer of the abdomen One 2^nd ^dr with sarcoma
4002, 44	One 1^st ^dr, age 51 Two 2^nd ^dr, age 57, 60 One 3^rd ^dr	
4042, 50	Two 1^st ^dr, age 37, 53 One 2^nd ^dr, age 55	One 2^nd ^dr with pancreatic cancer
5031, 46	One 1^st ^dr, age 44	
5611, 30	Two 2^nd ^dr, age 58, unknown One 3^rd ^dr, age 51	One 2^nd ^dr with cancer uteri

## Discussion

We have evaluated the prevalence of the CHEK2 1100delC variant in two cohorts of breast cancer patients from the Stockholm region, one familial and one population-based, both with a well-defined family history and in controls. In our study CHEK2 1100delC was associated with familial breast cancer (2.2% vs. 0.7% in controls, p = 0.03) and confirm previous results of the variant as a risk factor for breast cancer.

In the original studies from Finland and the Netherlands the prevalence in familial breast cancer was 3.1–5.5 % [7,8]. The highest prevalence of CHEK2 1100delC has been reported in familial non-*BRCA1/2 *families also harboring colon cancer cases (18%) and in some highly selected high-risk breast cancer families (9–11%) [9,15,16]. The prevalence varies according to ethnicity and in studies mainly from Central and southern Europe, the variant, even in high-risk families, is very rare (<1%) [17-20].

Sporadic breast cancer cases have been reported to have a higher prevalence of the variant than healthy controls as demonstrated in the large pooled study by the Breast Cancer Consortium (1,9% vs. 0.75%) [10]. In our study, we found no accumulation of *CHEK2 *1100delC among sporadic cases, the cohort however too small to draw any firm conclusions. The difference in prevalence in our two cohorts regarding familial breast cancer (1.9% vs. 2.9%) is also based on very few cases and we refrain from analyzing this difference.

The prevalence of *CHEK2 *1100delC in our controls was 0.7%, which is consistent with previous studies in the Swedish population (0.4–1%) and the large pooled control material of more than 9000 controls, mainly of Western European origin (0.7%) [10,21-23].

In our material the *CHEK2 *1100delC carriers were markedly younger at diagnosis compared to non-carriers, even though the difference was of borderline significance in the two groups of breast cancer cases and the material small. Several previous studies support this finding including the large pooled analysis where mutation prevalence decreased with increasing age at diagnosis [9,10,15,24-26]. Other studies have found a modest, non-significant, difference regarding age at onset and there are also negative studies including the original Dutch study [7,8,16,27,28]. In the Dutch study, however, only familial cases were included and the mean age in both carriers and non-carriers was 45 years, which was much lower than in breast cancer patients in general, resulting in decreased power for identification of differences in age at onset. In a recent Swedish study on postmenopausal breast cancer there was 0.7% 1100delC carriers in cases compared to 0.4% in controls, which is consistent with our data on older patients even with a family history (Table 2) since the majority of our mutation carriers were diagnosed premenopausally [23].

*CHEK2 *was originally suggested to be a high-risk gene for the Li Fraumeni syndrome [29]. There is limited or no co-segregation of CHEK2 1100delC and disease in families both in our and other previous studies which contradicts this role of CHEK2 [7-9,16,30,31]. The role for the CHEK2 1100delC variant might then either be a low risk variant on its own, or constitute a modifier of risk in syndromes with as yet unknown high-risk gene(s).

Our results, generated in a material with a well-defined family history including the paternal side, support the role of a modifier, as there was no accumulation of the variant in true sporadic cases, and the variant seemed to influence the age at diagnosis in carriers. Our material is however relatively small, and this conclusion would need to be verified in a larger material.

## Conclusion

In conclusion the CHEK2 1100delC variant was significantly more frequent in familial cases assumed to modify an underlying hereditary fault Since the effect of the variant is modest and the variant rare, there is no need for CHEK2 1100delC screening at present but the variant might prove interesting in combination with other genetic/non-genetic factors in the future.

## Competing interests

The author(s) declare that they have no competing interests.

## Authors' contributions

SM collected the population-based material, participated in the design of the study, performed the statistical analysis and wrote the manuscript.

HE participated in development of the method and in the molecular genetic analysis.

AL conceived the idea of the study, collected the familial risk cohort material and participated in the analysis of the study.

MLB participated in the design of the study, performed the molecular genetic analysis, and participated in the analysis of the results and in drafting the manuscript.

All authors read and approved the final manuscript

## Pre-publication history

The pre-publication history for this paper can be accessed here:



## References

[B1] Easton DF (1999). How many more breast cancer predisposition genes are there?. Breast Cancer Res.

[B2] Antoniou AC, Pharoah PD, McMullan G, Day NE, Stratton MR, Peto J, Ponder BJ, Easton DF (2002). A comprehensive model for familial breast cancer incorporating BRCA1, BRCA2 and other genes. Br J Cancer.

[B3] Pharoah PD, Antoniou A, Bobrow M, Zimmern RL, Easton DF, Ponder BA (2002). Polygenic susceptibility to breast cancer and implications for prevention. Nat Genet.

[B4] Dunning AM, Healey CS, Pharoah PD, Teare MD, Ponder BA, Easton DF (1999). A systematic review of genetic polymorphisms and breast cancer risk. Cancer Epidemiol Biomarkers Prev.

[B5] Antoniou AC, Easton DF (2003). Polygenic inheritance of breast cancer: Implications for design of association studies. Genet Epidemiol.

[B6] Bartek J, Lukas J (2003). Chk1 and Chk2 kinases in checkpoint control and cancer. Cancer Cell.

[B7] Vahteristo P, Bartkova J, Eerola H, Syrjakoski K, Ojala S, Kilpivaara O, Tamminen A, Kononen J, Aittomaki K, Heikkila P, Holli K, Blomqvist C, Bartek J, Kallioniemi OP, Nevanlinna H (2002). A CHEK2 genetic variant contributing to a substantial fraction of familial breast cancer. Am J Hum Genet.

[B8] Meijers-Heijboer H, van den Ouweland A, Klijn J, Wasielewski M, de Snoo A, Oldenburg R, Hollestelle A, Houben M, Crepin E, van Veghel-Plandsoen M, Elstrodt F, van Duijn C, Bartels C, Meijers C, Schutte M, McGuffog L, Thompson D, Easton D, Sodha N, Seal S, Barfoot R, Mangion J, Chang-Claude J, Eccles D, Eeles R, Evans DG, Houlston R, Murday V, Narod S, Peretz T, Peto J, Phelan C, Zhang HX, Szabo C, Devilee P, Goldgar D, Futreal PA, Nathanson KL, Weber B, Rahman N, Stratton MR (2002). Low-penetrance susceptibility to breast cancer due to CHEK2(*)1100delC in noncarriers of BRCA1 or BRCA2 mutations. Nat Genet.

[B9] Oldenburg RA, Kroeze-Jansema K, Kraan J, Morreau H, Klijn JG, Hoogerbrugge N, Ligtenberg MJ, van Asperen CJ, Vasen HF, Meijers C, Meijers-Heijboer H, de Bock TH, Cornelisse CJ, Devilee P (2003). The CHEK2*1100delC variant acts as a breast cancer risk modifier in non-BRCA1/BRCA2 multiple-case families. Cancer Res.

[B10] (2004). CHEK2*1100delC and susceptibility to breast cancer: a collaborative analysis involving 10,860 breast cancer cases and 9,065 controls from 10 studies. Am J Hum Genet.

[B11] Lindblom A (1993). A molecular study on familial breast cancer. Departments of Clinical Genetics and Oncology, Karolinska Hospital.

[B12] Zelada-Hedman M, Wasteson Arver B, Claro A, Chen J, Werelius B, Kok H, Sandelin K, Hakansson S, Andersen TI, Borg A, Borresen Dale AL, Lindblom A (1997). A screening for BRCA1 mutations in breast and breast-ovarian cancer families from the Stockholm region. Cancer Res.

[B13] Chen J, Hedman MZ, Arver BW, Sigurdsson S, Eyfjord JE, Lindblom A (1998). BRCA2 germline mutations in Swedish breast cancer families. Eur J Hum Genet.

[B14] Margolin S, Werelius B, Fornander T, Lindblom A (2004). BRCA1 mutations in a population-based study of breast cancer in Stockholm County. Genet Test.

[B15] Mateus Pereira LH, Sigurdson AJ, Doody MM, Pineda MA, Alexander BH, Greene MH, Struewing JP (2004). CHEK2:1100delC and female breast cancer in the United States. Int J Cancer.

[B16] Meijers-Heijboer H, Wijnen J, Vasen H, Wasielewski M, Wagner A, Hollestelle A, Elstrodt F, van den Bos R, de Snoo A, Fat GT, Brekelmans C, Jagmohan S, Franken P, Verkuijlen P, van den Ouweland A, Chapman P, Tops C, Moslein G, Burn J, Lynch H, Klijn J, Fodde R, Schutte M (2003). The CHEK2 1100delC mutation identifies families with a hereditary breast and colorectal cancer phenotype. Am J Hum Genet.

[B17] Offit K, Pierce H, Kirchhoff T, Kolachana P, Rapaport B, Gregersen P, Johnson S, Yossepowitch O, Huang H, Satagopan J, Robson M, Scheuer L, Nafa K, Ellis N (2003). Frequency of CHEK2*1100delC in New York breast cancer cases and controls. BMC Med Genet.

[B18] Jekimovs CR, Chen X, Arnold J, Gatei M, Richard DJ, Spurdle AB, Khanna KK, Chenevix-Trench G (2005). Low frequency of CHEK2 1100delC allele in Australian multiple-case breast cancer families: functional analysis in heterozygous individuals. Br J Cancer.

[B19] Kleibl Z, Novotny J, Bezdickova D, Malik R, Kleiblova P, Foretova L, Petruzelka L, Ilencikova D, Cinek P, Pohlreich P (2005). The CHEK2 c.1100delC germline mutation rarely contributes to breast cancer development in the Czech Republic. Breast Cancer Res Treat.

[B20] Osorio A, Rodriguez-Lopez R, Diez O, de la Hoya M, Ignacio Martinez J, Vega A, Esteban-Cardenosa E, Alonso C, Caldes T, Benitez J (2004). The breast cancer low-penetrance allele 1100delC in the CHEK2 gene is not present in Spanish familial breast cancer population. Int J Cancer.

[B21] Wagenius M, Borg A, Johansson L, Giwercman A, Bratt O (2006). CHEK2*1100delC is not an important high-risk gene in families with hereditary prostate cancer in southern Sweden. Scand J Urol Nephrol.

[B22] Isinger A, Bhat M, Borg A, Nilbert M (2006). CHEK2 1100delC in patients with metachronous cancers of the breast and the colorectum. BMC Cancer.

[B23] Einarsdottir K, Humphreys K, Bonnard C, Palmgren J, Iles MM, Sjolander A, Li Y, Chia KS, Liu ET, Hall P, Liu J, Wedren S (2006). Linkage disequilibrium mapping of CHEK2: common variation and breast cancer risk. PLoS Med.

[B24] de Bock GH, Schutte M, Krol-Warmerdam EM, Seynaeve C, Blom J, Brekelmans CT, Meijers-Heijboer H, van Asperen CJ, Cornelisse CJ, Devilee P, Tollenaar RA, Klijn JG (2004). Tumour characteristics and prognosis of breast cancer patients carrying the germline CHEK2*1100delC variant. J Med Genet.

[B25] Rashid MU, Jakubowska A, Justenhoven C, Harth V, Pesch B, Baisch C, Pierl CB, Bruning T, Ko Y, Benner A, Wichmann HE, Brauch H, Hamann U (2005). German populations with infrequent CHEK2*1100delC and minor associations with early-onset and familial breast cancer. Eur J Cancer.

[B26] Weischer M, Bojesen SE, Tybjaerg-Hansen A, Axelsson CK, Nordestgaard BG (2007). Increased risk of breast cancer associated with CHEK2*1100delC. J Clin Oncol.

[B27] de Jong MM, Nolte IM, Te Meerman GJ, van der Graaf WT, Oosterom E, Bruinenberg M, Steege G, Oosterwijk JC, van der Hout AH, Boezen HM, Schaapveld M, Kleibeuker JH, de Vries EG (2005). No increased susceptibility to breast cancer from combined CHEK2 1100delC genotype and the HLA class III region risk factors. Eur J Cancer.

[B28] Kilpivaara O, Bartkova J, Eerola H, Syrjakoski K, Vahteristo P, Lukas J, Blomqvist C, Holli K, Heikkila P, Sauter G, Kallioniemi OP, Bartek J, Nevanlinna H (2005). Correlation of CHEK2 protein expression and c.1100delC mutation status with tumor characteristics among unselected breast cancer patients. Int J Cancer.

[B29] Bell DW, Varley JM, Szydlo TE, Kang DH, Wahrer DC, Shannon KE, Lubratovich M, Verselis SJ, Isselbacher KJ, Fraumeni JF, Birch JM, Li FP, Garber JE, Haber DA (1999). Heterozygous germ line hCHK2 mutations in Li-Fraumeni syndrome. Science.

[B30] Dufault MR, Betz B, Wappenschmidt B, Hofmann W, Bandick K, Golla A, Pietschmann A, Nestle-Kramling C, Rhiem K, Huttner C, von Lindern C, Dall P, Kiechle M, Untch M, Jonat W, Meindl A, Scherneck S, Niederacher D, Schmutzler RK, Arnold N (2004). Limited relevance of the CHEK2 gene in hereditary breast cancer. Int J Cancer.

[B31] Sodha N, Houlston RS, Bullock S, Yuille MA, Chu C, Turner G, Eeles RA (2002). Increasing evidence that germline mutations in CHEK2 do not cause Li-Fraumeni syndrome. Hum Mutat.

